# Supplementary material for: Greater effects of mutual cooperation and defection on subsequent cooperation in direct reciprocity games than generalized reciprocity games: Behavioral experiments and analysis using multilevel models
Source: PLoS One. 2020 Nov 19;15(11):e0242607. doi: 10.1371/journal.pone.0242607 (PMC7676727; doi:10.1371/journal.pone.0242607)
Supplement: S1 Fig — (PDF) [file pone.0242607.s001.pdf]

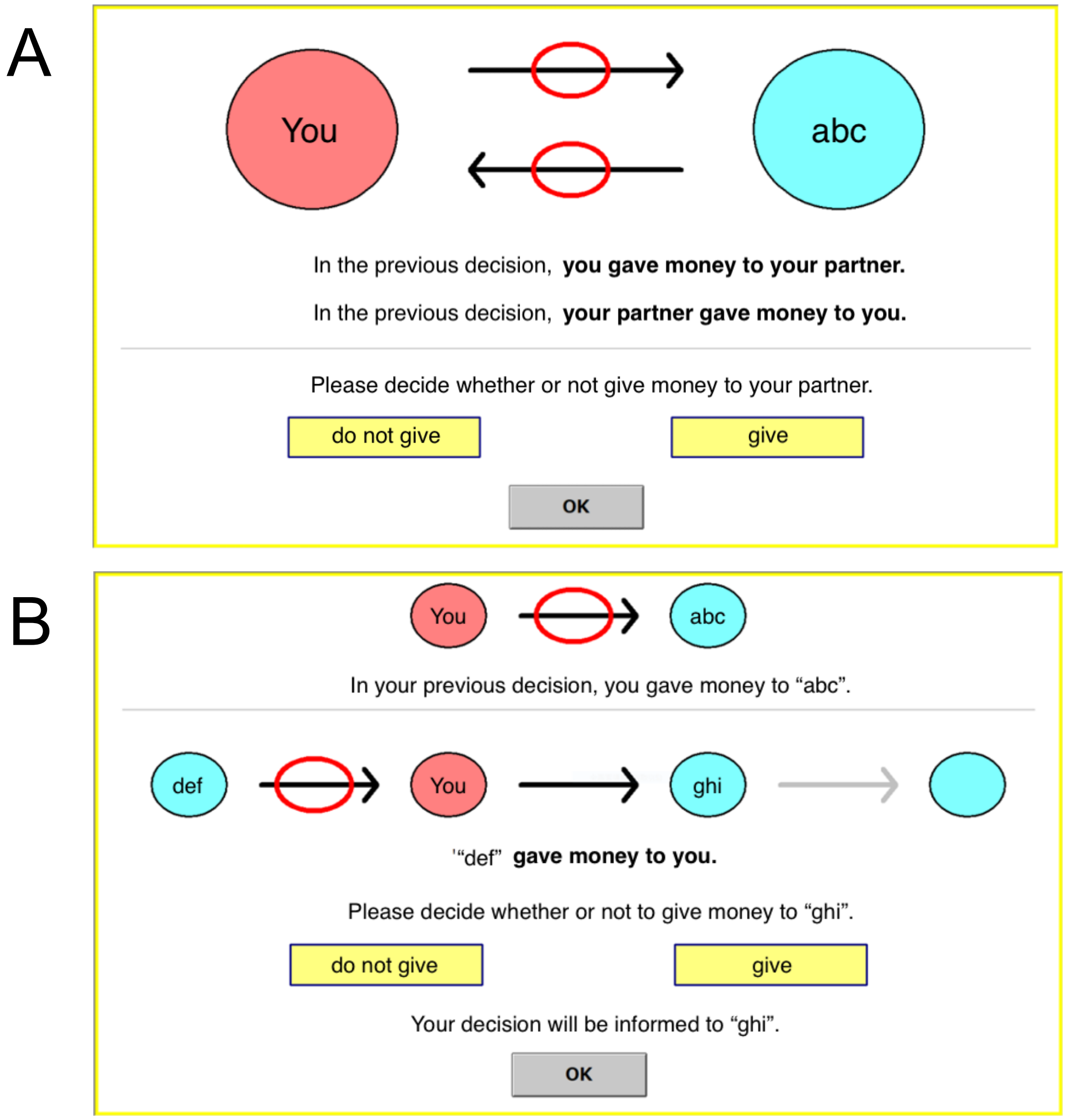

S1 Fig. Samples of the decision screens displayed to the participants. (A) Direct reciprocity game. (B) Generalized reciprocity game. In the experiments, the captions on the screen were displayed to the participants in Japanese.
