# Supplementary material for: Greater effects of mutual cooperation and defection on subsequent cooperation in direct reciprocity games than generalized reciprocity games: Behavioral experiments and analysis using multilevel models
Source: PLoS One. 2020 Nov 19;15(11):e0242607. doi: 10.1371/journal.pone.0242607 (PMC7676727; doi:10.1371/journal.pone.0242607)
Supplement: S2 Fig — (PDF) [file pone.0242607.s002.pdf]

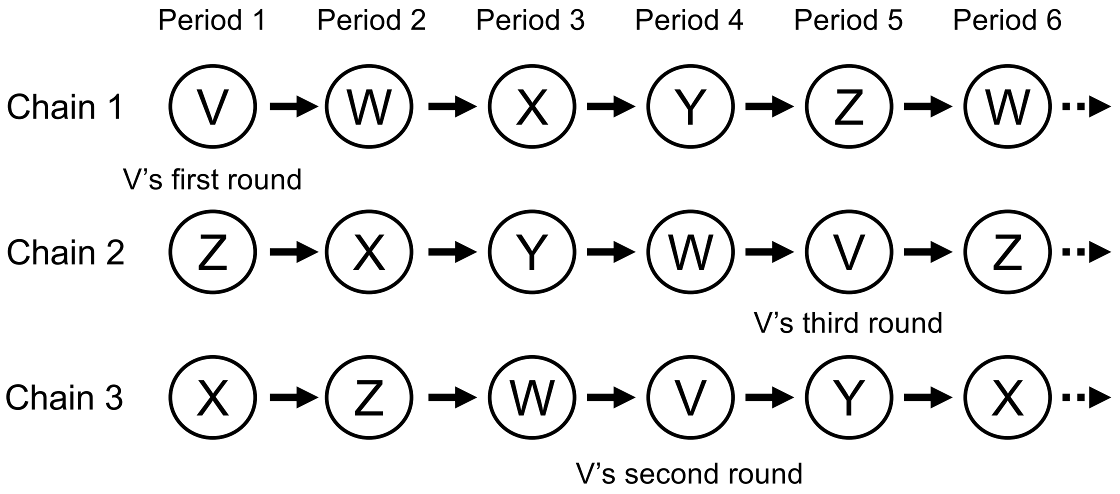

S2 Fig. Example of simultaneously running three independent chains of decisions in the generalized reciprocity game. To shorten the waiting time for the participants and reduce user fatigue, three chains of decisions were run simultaneously. Each participant was assigned the position of either V, W, X, Y, or Z in each of the three chains. Each position was arranged in such a way that no overlap with each other could occur in the same period. In a single period, each of the three players decided whether or not to donate money to each of their downstream neighbors. After all of them had submitted their decisions, the next period was started. For example, in the first period, players V, Z, and X simultaneously submitted their decisions. After they made their decisions, players W, X, and Z simultaneously submitted their decisions. The name of each player was displayed as a three-letter pseudonym on the computer screens (S1 Fig). Different pseudonyms were assigned for each chain of decisions; i.e., an assigned pseudonym was never used again in a single experimental session.
