# Supplementary material for: Greater effects of mutual cooperation and defection on subsequent cooperation in direct reciprocity games than generalized reciprocity games: Behavioral experiments and analysis using multilevel models
Source: PLoS One. 2020 Nov 19;15(11):e0242607. doi: 10.1371/journal.pone.0242607 (PMC7676727; doi:10.1371/journal.pone.0242607)
Supplement: S4 Fig — (PDF) [file pone.0242607.s004.pdf]

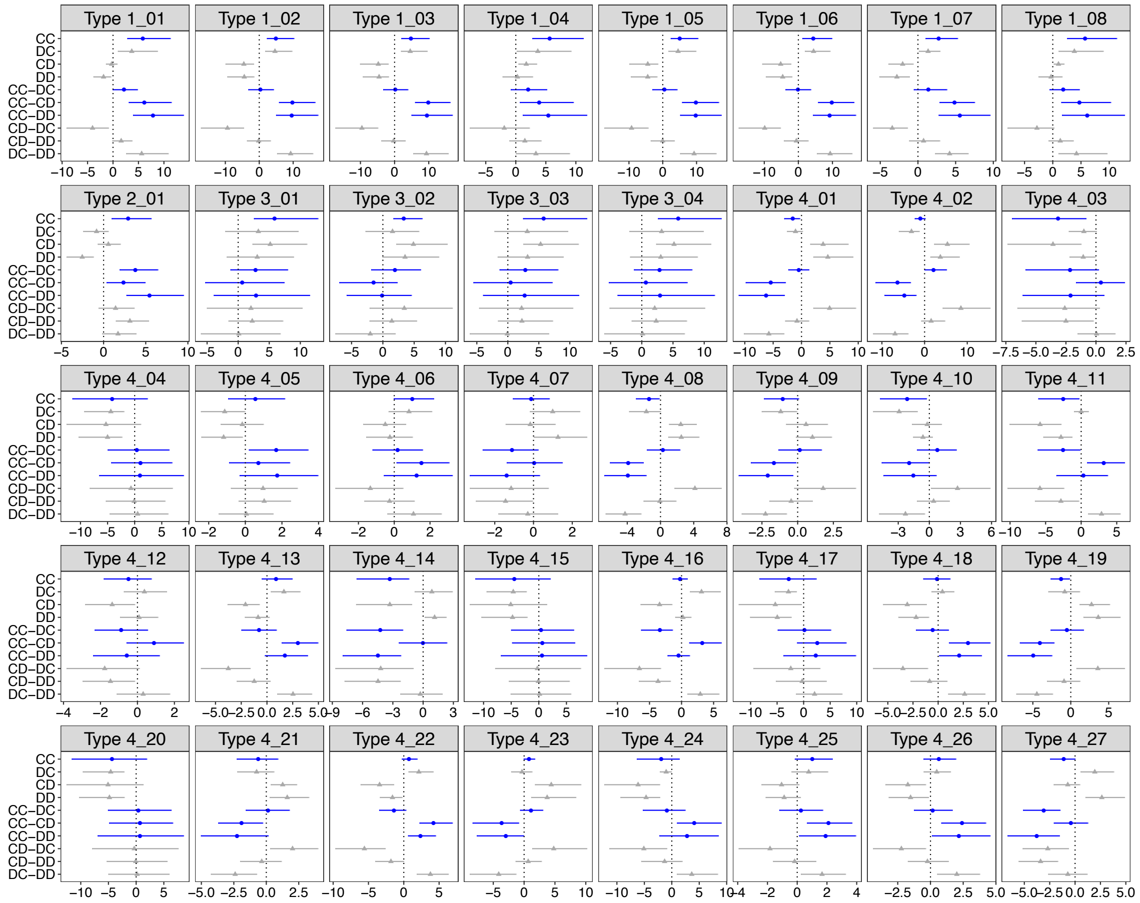

S4 Fig. Individual parameter values inferred separately for each participant in the generalized reciprocity game. Posterior distributions of the individual parameters in the own and partner's action model and the difference of predicted distributions between cooperation probabilities are shown separately for each participant. Behavioral types, classified according to the rule summarized in Table 2, are shown in each panel. The combinations of CC, DC, CD, and DD on the vertical axis represent the parameters that determine the cooperation probabilities: CC, $\beta_{1,i} + \beta_{2,i} + \beta_{3,i} + \beta_{4,i}$; DC, $\beta_{1,i} + \beta_{3,i}$; CD, $\beta_{1,i} + \beta_{2,i}$; and DD, $\beta_{1,i}$. If a value is greater than zero, the cooperation probability p is greater than 50% (i.e., $p = \exp(\beta)/(1+\exp(\beta))$). The combinations of CC–DC, CC–CD, CC–DD, CD–DC, CD–DD, and CD–DD on the vertical axis represent the inferred difference between cooperation probabilities; for example, CC–DC represents $\hat{p}(\mathrm{C}|\mathrm{CC})_i - \hat{p}(\mathrm{C}|\mathrm{DC})_i$.
