# Supplementary material for: Greater effects of mutual cooperation and defection on subsequent cooperation in direct reciprocity games than generalized reciprocity games: Behavioral experiments and analysis using multilevel models
Source: PLoS One. 2020 Nov 19;15(11):e0242607. doi: 10.1371/journal.pone.0242607 (PMC7676727; doi:10.1371/journal.pone.0242607)
Supplement: S5 Fig — (PDF) [file pone.0242607.s005.pdf]

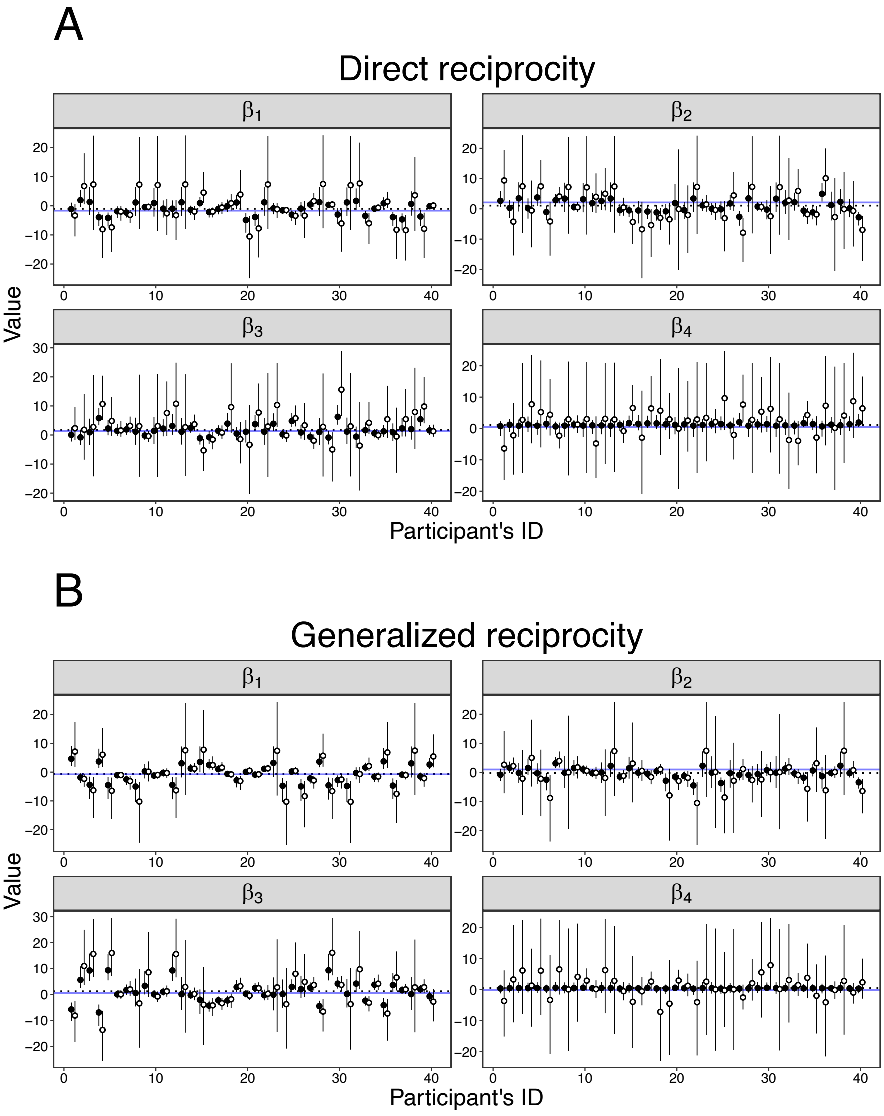

S5 Fig. Individual parameter values inferred by the multilevel, non-pooling, and pooling own and partner's action (OPA) model. (A) Direct reciprocity game. (B) Generalized reciprocity game. The vertical and horizontal axes indicate the parameter value and each participant, respectively. The closed and open circles represent the median of the participant's parameter inferred by the multilevel OPA model and the non-pooling OPA model, respectively. Each error bar represents the 95% compatibility interval of each parameter. The horizontal solid and dotted lines in each panel represent the median of the group-level parameter inferred by the pooling OPA model and that inferred by the multilevel OPA model.
