# Supplementary material for: Greater effects of mutual cooperation and defection on subsequent cooperation in direct reciprocity games than generalized reciprocity games: Behavioral experiments and analysis using multilevel models
Source: PLoS One. 2020 Nov 19;15(11):e0242607. doi: 10.1371/journal.pone.0242607 (PMC7676727; doi:10.1371/journal.pone.0242607)
Supplement: S1 Table — (PDF) [file pone.0242607.s006.pdf]

S1 Table. Posterior distributions of the parameters for each model in the direct reciprocity game.

Direct reciprocity game							
				Quantiles of posterior distribution			
Model	Parameter	Mean	SD	2.5%	50%	97.5%	ESS
Partner's action (PA)	v	1.14	0.38	0.44	1.12	1.92	14298
	$\mu_{\alpha 1}$	−0.10	0.55	−1.18	−0.11	1.05	1523
	$\mu_{\alpha 2}$	2.14	0.46	1.27	2.13	3.10	2679
	$\sigma_{\alpha 1}$	3.22	0.53	2.34	3.17	4.44	2960
	$\sigma_{\alpha 2}$	2.19	0.41	1.50	2.16	3.12	3684
Own and partner's action (OPA)	v	1.13	0.37	0.43	1.12	1.90	21345
	$\mu_{\beta 1}$	−1.00	0.57	−2.07	−1.02	0.19	1667
	$\mu_{\beta 2}$	1.10	0.63	−0.04	1.06	2.43	1948
	$\mu_{\beta 3}$	1.66	0.58	0.54	1.66	2.85	4032
	$\mu_{\beta 4}$	1.14	0.54	0.02	1.15	2.15	5656
	$\sigma_{\beta 1}$	2.55	0.55	1.63	2.49	3.79	2709
	$\sigma_{\beta 1}$	2.53	0.59	1.54	2.47	3.85	1827
	$\sigma_{\beta 3}$	2.41	0.45	1.63	2.38	3.41	4396
	$\sigma_{\beta 4}$	0.93	0.61	0.04	0.86	2.28	2866
Own action (OA)	v	1.14	0.39	0.41	1.13	1.93	19425
	$\mu_{\gamma 1}$	−0.04	0.44	−0.84	−0.07	0.87	1315
	$\mu_{\gamma 2}$	1.35	0.45	0.57	1.31	2.36	1454
	$\sigma_{\gamma 1}$	1.97	0.42	1.30	1.92	2.91	1949
	$\sigma_{\gamma 2}$	1.75	0.43	1.05	1.70	2.73	1454
Null	μ_{ε}	0.93	0.44	0.11	0.91	1.84	717
	σ_{ε}	2.69	0.41	2.00	2.65	3.58	1853

The posterior distributions are summarized by the mean, standard deviation (SD), and quantiles (2.5%, 50%, and 97.5%, respectively) for each parameter. ESS is the effective sample size.
