# Supplementary material for: Greater effects of mutual cooperation and defection on subsequent cooperation in direct reciprocity games than generalized reciprocity games: Behavioral experiments and analysis using multilevel models
Source: PLoS One. 2020 Nov 19;15(11):e0242607. doi: 10.1371/journal.pone.0242607 (PMC7676727; doi:10.1371/journal.pone.0242607)
Supplement: S2 Table — (PDF) [file pone.0242607.s007.pdf]

S2 Table. Posterior distributions of the parameters for each model in the generalized reciprocity game.

Generalized reciprocity game							
				Quantiles of posterior distribution			
Model	Parameter	Mean	SD	2.5%	50%	97.5%	ESS
Partner's action (PA)	v	0.93	0.47	0.04	0.91	1.88	10368
	$\mu_{\alpha 1}$	−0.49	0.60	−1.69	−0.48	0.70	776
	$\mu_{\alpha 2}$	1.23	0.71	−0.16	1.22	2.66	828
	$\sigma_{\alpha 1}$	3.51	0.61	2.52	3.44	4.88	2195
	$\sigma_{\alpha 2}$	4.05	0.65	3.00	3.98	5.51	1682
Own and partner's action (OPA)	v	0.75	0.34	0.11	0.74	1.42	24430
	$\mu_{\beta 1}$	−0.63	0.59	−1.80	−0.63	0.55	1989
	$\mu_{\beta 2}$	−0.25	0.54	−1.30	−0.25	0.83	2827
	$\mu_{\beta 3}$	1.23	0.77	−0.29	1.23	2.76	2345
	$\mu_{\beta 4}$	0.48	0.41	−0.31	0.48	1.29	13522
	$\sigma_{\beta 1}$	3.40	0.61	2.39	3.34	4.74	4139
	$\sigma_{\beta 1}$	2.31	0.53	1.45	2.25	3.52	3086
	$\sigma_{\beta 3}$	4.37	0.69	3.21	4.31	5.89	3715
	$\sigma_{\beta 4}$	0.50	0.40	0.02	0.41	1.49	5579
Own action (OA)	v	0.75	0.35	0.08	0.74	1.44	25095
	$\mu_{\gamma 1}$	−0.10	0.31	−0.70	−0.11	0.53	1636
	$\mu_{\gamma 2}$	0.01	0.37	−0.69	−0.01	0.80	2453
	$\sigma_{\gamma 1}$	1.73	0.32	1.20	1.69	2.46	2827
	$\sigma_{\gamma 2}$	1.71	0.39	1.09	1.66	2.62	2021
Null	μ_{ε}	0.03	0.33	−0.61	0.02	0.67	520
	σ_{ε}	2.01	0.30	1.51	1.98	2.69	1777

The posterior distributions are summarized by the mean, standard deviation (SD), and quantiles (2.5%, 50%, and 97.5%, respectively) for each parameter. ESS is the effective sample size.
