# Supplementary material for: Greater effects of mutual cooperation and defection on subsequent cooperation in direct reciprocity games than generalized reciprocity games: Behavioral experiments and analysis using multilevel models
Source: PLoS One. 2020 Nov 19;15(11):e0242607. doi: 10.1371/journal.pone.0242607 (PMC7676727; doi:10.1371/journal.pone.0242607)
Supplement: S3 Table — (PDF) [file pone.0242607.s008.pdf]

S3 Table. WAIC values for the multilevel, non-pooling, and pooling own and partner's action (OPA) model.

	WAIC	p_{WAIC}	dWAIC	SE	dSE	weight
Direct reciprocity game						
Multilevel OPA	1113.02	76.17	0	45.33	NA	0.65
Non-pooling OPA	1114.26	105.35	1.24	51.08	13.26	0.35
Pooling OPA	1649.87	5.07	536.85	43.92	38.37	0
Generalized reciprocity game						
Multilevel OPA	1238.66	77.92	0	43.28	NA	1
Non-pooling OPA	1259.64	109.41	20.97	50.18	12.40	0
Pooling OPA	2211.08	5.04	972.42	22.20	43.71	0

dWAIC = difference between the WAIC of each model and that of the best model (i.e., the OPA model), SE = standard error of each WAIC, dSE = standard error of the dWAIC, and weight = the weight of the dWAIC.
