# Supplementary material for: Greater effects of mutual cooperation and defection on subsequent cooperation in direct reciprocity games than generalized reciprocity games: Behavioral experiments and analysis using multilevel models
Source: PLoS One. 2020 Nov 19;15(11):e0242607. doi: 10.1371/journal.pone.0242607 (PMC7676727; doi:10.1371/journal.pone.0242607)
Supplement: S1 Text — (PDF) [file pone.0242607.s009.pdf]

Parameter inference for the multilevel models

Here, we introduce the details of the multilevel models used in the current research. A non-centered parameterization was considered when fitting the multilevel models to the data aiming to reduce the inefficiency of MCMC sampling and to obtain converged results [1]. The following Equation (S1) is presented as an example of a model in which the number of parameters and participants are denoted by *K* and *I*, respectively:

$$
\begin{gathered}
\boldsymbol{m}_i = \boldsymbol{\mu} + \begin{bmatrix} m'_{1,i} \\ m'_{2,i} \\ \vdots \\ m'_{K,i} \end{bmatrix}, \\
\begin{pmatrix} m'_{1,1} & m'_{1,2} & \cdots & m'_{1,I} \\ m'_{2,1} & m'_{2,2} & \cdots & m'_{2,I} \\ \vdots & \vdots & \ddots & \vdots \\ m'_{K,1} & m'_{K,2} & \cdots & m'_{K,I} \end{pmatrix} = \mathrm{diag}(\boldsymbol{\sigma})\, \boldsymbol{L}\, \boldsymbol{Z}, \\
\mathrm{diag}(\boldsymbol{\sigma}) = \begin{pmatrix} \sigma_1 & & & 0 \\ & \sigma_2 & & \\ & & \ddots & \\ 0 & & & \sigma_K \end{pmatrix}, \\
\boldsymbol{Z} = \begin{pmatrix} z_{1,1} & z_{1,2} & \cdots & z_{1,I} \\ z_{2,1} & z_{2,2} & \cdots & z_{2,I} \\ \vdots & \vdots & \ddots & \vdots \\ z_{K,1} & z_{K,2} & \cdots & z_{K,I} \end{pmatrix}, \\
\boldsymbol{L}\boldsymbol{L}^{\mathrm{T}} = \boldsymbol{\Omega}, \\
\boldsymbol{\Omega} = \begin{pmatrix} 1 & \rho_{1,2} & \cdots & \rho_{1,K} \\ \rho_{2,1} & 1 & \cdots & \rho_{2,K} \\ \vdots & \vdots & \ddots & \vdots \\ \rho_{K,1} & \rho_{K,2} & \cdots & 1 \end{pmatrix}, \\
\boldsymbol{\mu} = \begin{bmatrix} \mu_1 \\ \mu_2 \\ \vdots \\ \mu_K \end{bmatrix}, \\
\boldsymbol{\sigma} = \begin{bmatrix} \sigma_1 \\ \sigma_2 \\ \vdots \\ \sigma_K \end{bmatrix}, \\
\boldsymbol{\mu} \sim \mathrm{Normal}(0,10), \\
\boldsymbol{\sigma} \sim \mathrm{HalfCauchy}(0,1),
\end{gathered}
\tag{S1}
$$

$$\boldsymbol{Z} \sim \text{Normal}(0,1),$$
$$\boldsymbol{L} \sim \text{LKJcorr\_Cholesky}(2),$$

where $\boldsymbol{\sigma}$, $\boldsymbol{\Omega}$, and ρ represent the vector of the standard deviation (SD) of varying effects, correlation matrix, and correlation coefficient between the parameters, respectively. $\boldsymbol{L}$ is a Cholesky factor of the correlation matrix of parameters, $\boldsymbol{\Omega}$: $\boldsymbol{\Omega} = \boldsymbol{L}\boldsymbol{L}^{\mathrm{T}}$. $\boldsymbol{Z}$ represents the z-scores matrix of the parameters for each participant. Multiplying a diagonal matrix of vector $\boldsymbol{\sigma}$ (group-level variance) by the Cholesky factor of the correlation matrix, $\boldsymbol{L}$ produced a Cholesky factor for the covariance matrix. Then, multiplying the Cholesky factorized covariance matrix by $\boldsymbol{Z}$ and adding the vector of group-level mean $\boldsymbol{\mu}$ generated a vector of the individual effects for each participant $\boldsymbol{m}_i$.

Weakly informative priors were set for each parameter. Concerning the vector of the group-level mean parameter $\boldsymbol{\mu}$, the normal distribution with a mean of 0 and a SD of 10 was set as the priors. Half–Cauchy prior was applied to the vector of the variance parameters $\boldsymbol{\sigma}$. A prior of $\boldsymbol{L}$ was defined as the Cholesky factorized LKJ correlation distribution [2], with the shape parameter equal to 2. A prior of $\boldsymbol{Z}$ was defined as the normal distribution with a mean of 0 and a SD of 1.

1. Stan Development Team. "22. 7 Reparameterization" In *Stan User's Guide Version 2.22.* 2019; Available from: https://mc-stan.org/docs/2_22/stan-users-guide/reparameterization-section.html (Accessed: 9, April, 2020)
2. Lewandowski D, Kurowicka D, Joe H. Generating random correlation matrices based on vines and extended onion method. J Multivar Anal. 2009; 100(9): 1989–2001. doi: 10.1016/j.jmva.2009.04.008
