# Supplementary material for: Greater effects of mutual cooperation and defection on subsequent cooperation in direct reciprocity games than generalized reciprocity games: Behavioral experiments and analysis using multilevel models
Source: PLoS One. 2020 Nov 19;15(11):e0242607. doi: 10.1371/journal.pone.0242607 (PMC7676727; doi:10.1371/journal.pone.0242607)
Supplement: S2 Text — (PDF) [file pone.0242607.s010.pdf]

Method for calculating the cooperation probabilities

<u>Predicted distribution of cooperation</u>

As shown in Equation (4) presented in the main text, the probability of cooperation p was predicted using the OPA model as follows:

$$p = \frac{\exp(\beta)}{1 + \exp(\beta)},$$

$$\beta = \begin{cases} \beta_{1,i} + \beta_{2,i}O_{i,t-1} + \beta_{3,i}P_{i,t-1} + \beta_{4,i}O_{i,t-1}P_{i,t-1} \\ v \left(\text{if } O_{i,t-1} \text{ or } P_{i,t-1} \text{ is a missing value}\right) \end{cases},$$

$$\beta_{1,i} = \mu_{\beta 1} + z_{\beta 1,i}\sigma_{\beta 1}, \tag{4}$$

$$\beta_{2,i} = \mu_{\beta 2} + z_{\beta 2,i}\sigma_{\beta 2},$$

$$\beta_{3,i} = \mu_{\beta 3} + z_{\beta 3,i}\sigma_{\beta 3},$$

$$\beta_{4,i} = \mu_{\beta 4} + z_{\beta 4,i}\sigma_{\beta 4},$$

The group-level cooperative tendency conditioned according to both own and the partner's previous actions was predicted by the linear equation including two predictor variables (O and P) and four parameters ($\mu_{\beta 1}$, $\mu_{\beta 2}$, $\mu_{\beta 3}$, and $\mu_{\beta 4}$). Then, group-level probabilities of cooperation conditioned by own and the partner's previous actions were calculated as follows:

$$\beta_{\mathrm{CC}} = \mu_{\beta 1} + \mu_{\beta 2} + \mu_{\beta 3} + \mu_{\beta 4},$$

$$\beta_{\mathrm{DC}} = \mu_{\beta 1} + \mu_{\beta 3},$$

$$\beta_{\mathrm{CD}} = \mu_{\beta 1} + \mu_{\beta 2},$$

$$\beta_{\mathrm{DD}} = \mu_{\beta 1},$$

$$\hat{p}(\mathrm{C|CC}) = \frac{\exp(\beta_{\mathrm{CC}})}{1 + \exp(\beta_{\mathrm{CC}})},$$

$$\hat{p}(\mathrm{C|DC}) = \frac{\exp(\beta_{\mathrm{DC}})}{1 + \exp(\beta_{\mathrm{DC}})}, \tag{S2}$$

$$\hat{p}(\mathrm{C|CD}) = \frac{\exp(\beta_{\mathrm{CD}})}{1 + \exp(\beta_{\mathrm{CD}})},$$

$$\hat{p}(\mathrm{C|DD}) = \frac{\exp(\beta_{\mathrm{DD}})}{1 + \exp(\beta_{\mathrm{DD}})}.$$

In a similar manner, concerning the PA model, the probability of cooperation conditioned according to the partner's previous action was derived as follows:

$$\alpha_{\mathrm{C}} = \mu_{\alpha 1} + \mu_{\alpha 2},$$
$$\alpha_{\mathrm{D}} = \mu_{\alpha 1},$$
$$\hat{p}(\mathrm{C}|\mathrm{C}) = \frac{\exp(\alpha_{\mathrm{C}})}{1 + \exp(\alpha_{\mathrm{C}})}, \qquad \text{(S3)}$$
$$\hat{p}(\mathrm{C}|\mathrm{D}) = \frac{\exp(\alpha_{\mathrm{D}})}{1 + \exp(\alpha_{\mathrm{D}})}.$$

The cooperative tendency depending on both own and the partner's previous actions could be calculated separately by each participant. The individual cooperative probabilities, denoted by $\hat{p}(\mathrm{C}|\mathrm{CC})_i$, $\hat{p}(\mathrm{C}|\mathrm{DC})_i$, $\hat{p}(\mathrm{C}|\mathrm{CD})_i$, and $\hat{p}(\mathrm{C}|\mathrm{DD})_i$ in the main text, could be calculated using inferred values of each individual parameter, $\beta_{1,i}$, $\beta_{2,i}$, $\beta_{3,i}$, and $\beta_{4,i}$ instead of $\mu_{\beta 1}$, $\mu_{\beta 2}$, $\mu_{\beta 3}$, and $\mu_{\beta 4}$ in the Equation (S2).

Empirical probabilities of cooperation

Empirical probabilities of cooperation conditioned according to both own and partner's previous actions were calculated based on the experimental data. The probabilities were calculated by aggregating over all participants and separately by each participant. The probabilities were obtained as follows:

$$p(\mathrm{C}|\mathrm{CC}) = \frac{C_{\mathrm{CC}}}{N_{\mathrm{CC}}},$$
$$p(\mathrm{C}|\mathrm{DC}) = \frac{C_{\mathrm{DC}}}{N_{\mathrm{DC}}},$$
$$p(\mathrm{C}|\mathrm{CD}) = \frac{C_{\mathrm{CD}}}{N_{\mathrm{CD}}}, \qquad \text{(S4)}$$
$$p(\mathrm{C}|\mathrm{DD}) = \frac{C_{\mathrm{DD}}}{N_{\mathrm{DD}}},$$

where N and C represented the total number of cases and the number of times cooperation was selected, respectively. First and second subscript letter of N and C denoted own and partner's actions in the previous round, respectively (C = cooperation; D = defection). For example, N_{CD} represented the total number of the cases in which the player had cooperated, but he/she was informed about his/her partner's defection. C_{CD} represented the total number of the cases in which the player's cooperation was performed after the case in which the player cooperated, but the partner defected. Then, $p(\mathrm{C}|\mathrm{CD})$ denoted the empirical probability of cooperation after the player had

cooperated, and his/her partner had defected. Other probabilities were calculated in a similar way.

In a similar manner, empirical probabilities of cooperation conditioned by the partner's previous action, $p(\mathrm{C}|\mathrm{C})$ and $p(\mathrm{C}|\mathrm{D})$, were defined as follows:

$$p(\mathrm{C}|\mathrm{C}) = \frac{C_\mathrm{C}}{N_\mathrm{C}}, \qquad p(\mathrm{C}|\mathrm{D}) = \frac{C_\mathrm{D}}{N_\mathrm{D}}, \tag{S5}$$

where N_C and N_D represented the total number of cases in which the player experienced cooperation and defection from his/her partner, respectively. C_C and C_D represented the total number of cooperation occurred after the partner cooperated and defected, respectively.

Empirical cooperation probabilities were also calculated separately by each participant. It should be noted that the total number of each combination (N_CC, N_CD, N_DC, and N_DD) was different for each participant.
